# Beyond Anatomy: Use of Sinus Propagation Mapping to Identify the Slow Pathway for Cryoablation in Pediatric Patients

**DOI:** 10.19102/icrm.2023.14124

**Published:** 2023-12-15

**Authors:** William M. Fogarty IV, Anna N. Kamp, Mariah Eisner, Naomi J. Kertesz, Rohan N. Kumthekar

**Affiliations:** 1 Division of Cardiology, Nationwide Children’s Hospital, Columbus, OH, USA; 2 Department of Pediatrics, The Ohio State University College of Medicine, Columbus, OH, USA; 3 Center for Biostatistics, The Ohio State University College of Medicine, Columbus, OH, USA

**Keywords:** Ablation, arrhythmia mapping, atrioventricular nodal re-entrant tachycardia, pediatric electrophysiology, supraventricular tachycardia

## Abstract

Slow pathway modification via cryoablation is a common treatment of atrioventricular nodal re-entrant tachycardia (AVNRT) in pediatric patients. Sinus propagation mapping (SPM) is a tool that has been used to augment identification of the AVNRT slow pathway. We hypothesize that the use of SPM will decrease the total number of ablations performed and decrease the number of ablations until the slow pathway is successfully modified without a significant increase in procedure time. We conducted a retrospective review of patients who underwent cryoablation for AVNRT from August 2016 through March 2021. We excluded patients >21 years of age, those who underwent radiofrequency ablation; those with prior AVNRT ablation, additional pathways, or arrhythmias; and those with congenital heart disease. Out of 122 patients identified by the IMPACT database query, 103 met the inclusion criteria. Fifty-two patients (50.5%) had SPM completed during their procedures. The median number of ablations needed until successful slow pathway modification was two ablations in patients who underwent SPM and four ablations in the non-SPM group (*P* = .03). There was no significant difference in the total number of ablations between groups. The median total procedural time was longer in the SPM group (152 vs. 125 min; *P* = .01). SPM can be utilized to further improve the successful treatment of AVNRT with cryotherapy by lowering the number of ablations needed until successful slow pathway modification. However, the technique requires some additional time to collect sufficient data points to create the sinus map.

## Introduction

In the pediatric and young adult populations, atrioventricular nodal re-entrant tachycardia (AVNRT) is a common form of re-entrant supraventricular tachycardia (SVT) affecting 30%–50% of adolescent patients diagnosed with SVT.^1,2^ Patients with AVNRT have dual atrioventricular node physiology, with slow and fast conduction pathways, creating the substrate for AVNRT. Both pediatric and adult consensus guidelines recommend catheter ablation for the long-term management of symptomatic patients with AVNRT.^1,3,4^ Ablation has also become the preferred first-line treatment of choice from the standpoint of patients and families.^5,6^ Cryoablation has become a popular technique in the treatment of AVNRT in pediatrics due to its high acute success rate, lower risk of iatrogenic atrioventricular block, and improved catheter stability compared to the more traditional radiofrequency ablation due to cryoadherence to target cardiac tissue.^7–9^

The objective of cryoablation is to modify the slow pathway in the AVNRT circuit. To optimize the location of the slow pathway and procedural success, electrophysiologists have traditionally used anatomical landmarks with ideal intracardiac electrogram characteristics and voltage gradient mapping as tools to determine the area of the slow pathway.^7,10^ Although many slow pathways can be localized using voltage mapping, others do not have a contained low-voltage bridge, making it difficult to definitively know where to ablate.

**Figure 1:**
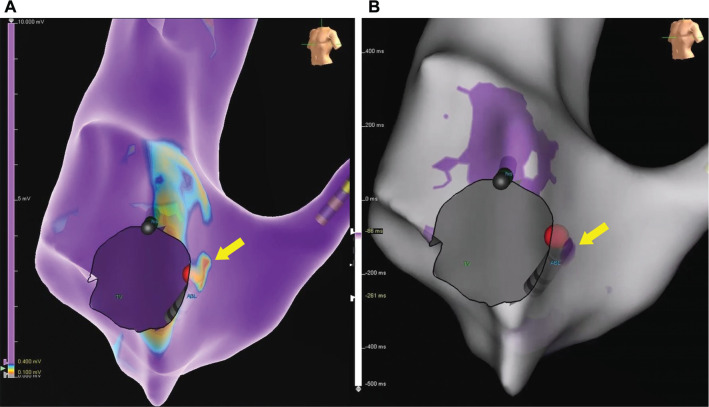
Electroanatomic and sinus propagation map. **A:** An example of a left anterior oblique view of the right atrial electroanatomic voltage map. Note the area of low voltage designated by the color gradient change from higher voltage (>0.4 mV) in purple to lower voltage in red (0.1 mV). The arrow highlights this area as the low-voltage bridge. The red sphere represents a successful site of cryotherapy. The tricuspid valve (TV) annulus, ablation catheter (ABL), and His catheter are labeled, while the coronary sinus catheter is positioned in the coronary sinus. **B:** Still frame from a sinus propagation map with the same geometry as seen in **(A)** from the same patient. The arrow highlights the site of wave collision seen in purple, which is similar in proximity to the low-voltage bridge seen in **(A)**. The red sphere represents a successful site of cryotherapy. The TV annulus, ABL, and His catheter are labeled. *Abbreviations:* ABL, ablation catheter; TV, tricuspid valve.

Sinus propagation mapping (SPM) is a newer tool that can be used to augment the identification of the slow pathway and improve cryoablation success.^2^ In 1990, Chang et al. first described computerized activation sequence mapping of the atrial septum. Their findings helped demonstrate the basis of and potential for ablation of the most common types of supraventricular arrhythmias, including AVNRT.^11^ Sinus mapping is performed by creating a propagation map of the atrial impulse in sinus rhythm. The area of activation where the wavefronts collide typically correlates with the location of the slow pathway,^2^ as seen in **Figure 1**. The mechanism by which the two correlate is still unclear, but it is most likely a coincidental surrogate marker.

The aim of this retrospective study was to evaluate the effect of SPM on cryoablation of AVNRT in pediatric patients. We hypothesized that the use of SPM would decrease the total number of ablations performed and decrease the number of ablations until the slow pathway was successfully modified without a significant increase in procedure time.

## Methods

The study was approved by the Nationwide Children’s Hospital Institutional Review Board under the study number 00001784. The need for informed consent was waived with approval from the institutional review board due to the retrospective nature of the study. Patients with AVNRT who underwent an electrophysiology study with cryoablation between August 2016 and March 2021 were identified through a query of an internal IMPACT database. Patients >21 years of age were excluded from the analysis along with those who had previous ablations performed; those who underwent radiofrequency ablation during their electrophysiology study, those with arrhythmia substrates other than AVNRT, and those with congenital heart disease were likewise excluded. In addition, patients without acute procedural success were also excluded from the statistical analysis.

Cryoablation is the preferred therapy for catheter modification of AVNRT at our institution. The few AVNRT patients treated with isolated or concomitant radiofrequency ablation were excluded from the analysis to improve the homogeneity of the data. Sinus mapping was completed using ablation catheters in a point-by-point manner. Multi-electrode catheters were not used during the mapping process. While other institutions may utilize cryomapping, our institution favors a different approach. Once AVNRT is reproducibly induced and the designated ablation site is determined, a cryotherapy lesion is initiated. Seventeen seconds after the temperature reaches <0°C, re-induction of AVNRT is attempted. If there is no inducible AVNRT and/or a change in the AV node effective refractory period, a full 4-min lesion is placed. The first successful lesion is also the site of a “freeze–thaw–freeze” double lesion. Markers of successful slow pathway modification include the elimination of sustained slow pathway conduction, no more than single re-entry beats following an A–H jump, and the absence of inducible AVNRT. Four additional “insurance” lesions are then placed around the initial site. If the initial markers of slow pathway modification remain met after a 30-min waiting period, the procedure is considered to be acutely successful. Prior to June 2018, SPM was not conducted on patients; SPM was only implemented into routine practice at the end of 2018, thus dividing the cohorts temporally. There were no other technical or procedural changes during the study period.

Basic demographic data were collected, including age at the time of the procedure, sex, height, weight, and body surface area (BSA). Details of the procedure, including the performing physician, the three-dimensional (3D) mapping system used, procedural date, the use of sinus mapping, the number of sinus mapping points, the presence of inducible AVNRT (typical or atypical) with or without the need for isoproterenol to induce SVT, the total procedure time, and complications, were logged. Ablation parameters, including the location of the slow pathway, the number of ablations needed until success was achieved, the total number of ablations performed (including test and complete lesions), the time of the first successful lesion, and the total cryotherapy time, were also recorded. The follow-up parameters measured included arrhythmia recurrence at the most recent follow-up date.

Acute procedural success was defined as the elimination of sustained slow pathway conduction, no more than single re-entry beats following an A–H jump, and the absence of inducible AVNRT. Slow pathway elimination was not routinely performed due to the lack of additional freedom from recurrence.^12^ Total procedural time was calculated from the time of the first catheter insertion to the time of the local anesthetic agent given. The first successful lesion was defined as the first cryoablation lesion that resulted in a change in the underlying substrate (either loss of inducible SVT or loss of A–H jump). All cases were performed using either the EnSite™ 3D mapping system (Abbott, Chicago, IL, USA) or Carto™ (Biosense Webster, Diamond Bar, CA, USA). There were no other technical or procedural changes during the reviewed time frame.

Variable distributions were evaluated using bar and violin plots. Data were summarized using frequencies (%) for categorical variables and median (interquartile range [IQR]) values for continuous variables. Group differences between subjects who did and did not receive SPM were assessed with the Wilcoxon rank-sum test, chi-squared test, or Fisher’s exact test. Recurrence-free survival analysis for AVNRT was performed using the Kaplan–Meier method; differences between the two groups were evaluated with the log-rank test. Two-sided *P*-values were reported at the .05 significance level. All statistical analyses were performed in R version 4.0 (R Foundation for Statistical Computing, Vienna, Austria) with reproducible programming in R Markdown.

## Results

**Figure 2:**
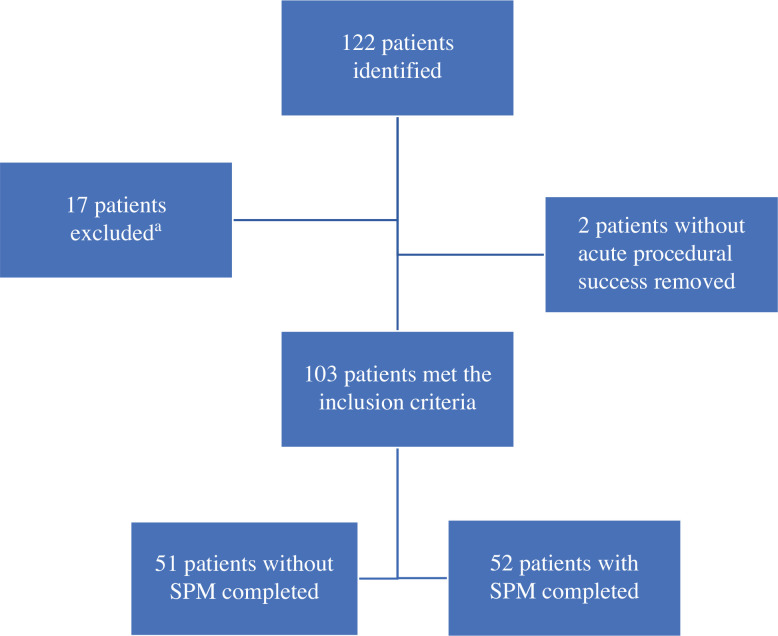
Patient identification flowchart. ^a^Excluded patients included 3 excluded for being >21 years of age at the time of the procedure, 10 excluded for repeat ablations, 3 excluded for having congenital heart disease, 6 excluded for using radiofrequency ablation, 2 excluded for having accessory pathway–mediated supraventricular tachycardia, and 1 excluded for having atrial fibrillation. Seven of the 17 patients met more than one exclusion criteria. *Abbreviation:* SPM, sinus propagation mapping.

Of 122 patients who underwent an electrophysiology study with documented AVNRT during the study period, 17 patients were excluded **(Figure 2)**. Also, 2 patients from the non-SPM group who did not have acute procedural success and were subsequently treated with medical therapy for their SVT were also excluded from the statistical analysis. The remaining 103 patients met the inclusion criteria and were separated into two groups: those who underwent SPM and those who did not. Of those 103 patients, 52 (50.5%) had SPM during their electrophysiology study. The SPM group had a median age of 15 years (IQR, 11–16 years) and a median weight of 57 kg (IQR, 44–69 kg), and 58% of the group was female. The non-SPM group had a median age of 14 years (IQR, 12–16 years) and a median weight of 55 kg (IQR, 49–60 kg), and 63% of the group was female. There were no statistically significant differences in age, sex, height, weight, or BSA between the SPM and non-SPM groups **(Table 1)**. There was also no statistical difference between the groups regarding the physician performing the procedure or the 3D mapping system used **(Table 1)**.

Inducible typical AVNRT occurred in 99 (96%) patients, with 4 (4%) patients having inducible atypical “fast–slow” AVNRT. Isoproterenol was used for either AVNRT induction or testing in 96 (93%) patients. There was no difference in AVNRT induction between the SPM and non-SPM groups (*P* = .9) **(Table 2)**. The median number of sinus mapping points for the SPM group was 411 (IQR, 179–581).

The median number of ablation lesions until successful slow pathway modification was lower in the SPM group than in the non-SPM group (2 vs. 4; *P* = .026) **(Table 2)**. There was no difference in the total number of ablations performed between the two groups, each with a median of 11 ablations (SPM IQR, 8–16; non-SPM IQR, 7–15; *P* ≥ .9). However, 38% of the sinus mapping group (n = 20) had successful slow pathway modification on the first ablation lesion compared to 16% of the non–sinus mapping group (n = 8) (*P* = .009). Total procedural time and time until the first successful lesion were longer for the SPM group compared to the non-SPM group, with median durations of 153 (IQR, 134–179) versus 125 (IQR, 107–164) min (*P* = .003) and 69 (IQR, 60–90) versus 52 (IQR, 40–83) min (*P* = .012), respectively. Total cryoablation application time did not differ significantly between the two groups, with median times of 33 (IQR, 25–46) min for the SPM group and 28 (IQR, 24–37) min for the non-SPM group (*P* = .2). There were no procedural complications noted in either group.

**Figure 3:**
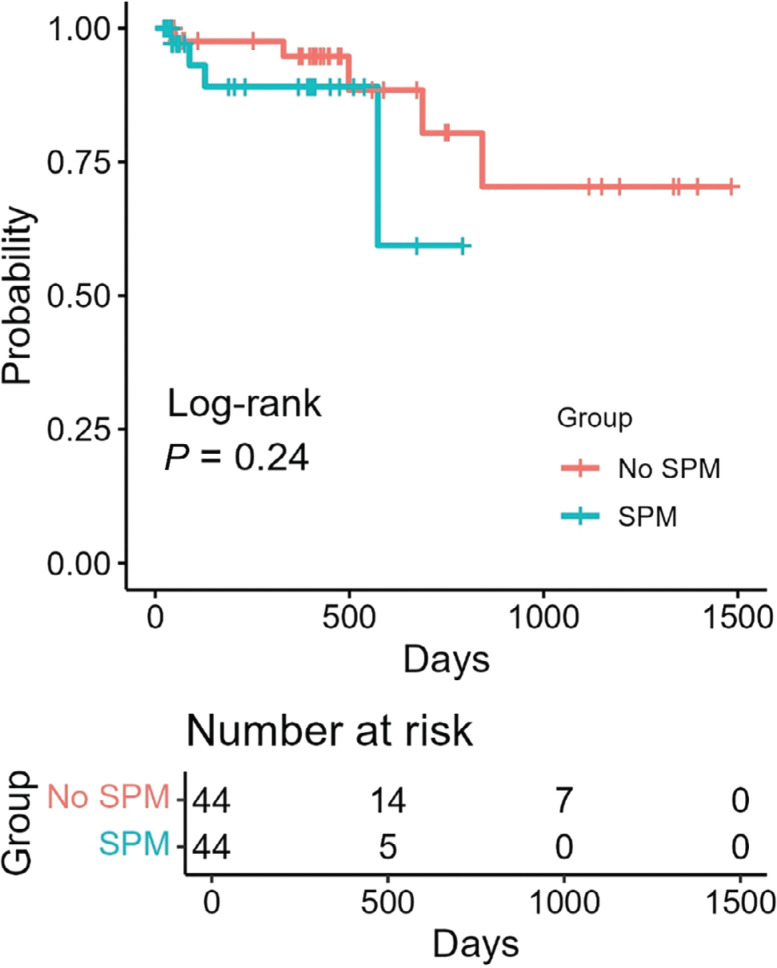
Kaplan–Meier curve of recurrence-free survival of supraventricular tachycardia with and without SPM. *Abbreviation:* SPM, sinus propagation mapping.

A total of 88 subjects had sufficient follow-up data, including 44 subjects in each group. The median follow-up durations were 446 (IQR, 338–836) days for the non-SPM group and 158 (IQR, 42–407) days for the SPM group. In total, there were nine cases of SVT recurrence (8.7%) between the two groups (5 cases in the non-SPM cohort and 4 cases in the SPM cohort). The median time until documented recurrence in the non-SPM cohort was 498 (IQR, 330–688) days, while that in the SPM cohort was 108 (IQR, 76–239) days **(Table 2)**. A Kaplan–Meier survival analysis demonstrated no difference in time to SVT recurrence between the two groups **(Figure 3)**.

## Discussion

When comparing these two demographically similar cohorts, the addition of SPM significantly lowered the number of cryoablations needed to successfully modify the AVNRT slow pathway. However, this improved precision of slow pathway localization came at the expense of increased total procedural time and time until the first successful ablation was performed. The additional time spent completing the mapping process, such as time spent collecting the mapping points (median, 411 points), likely accounts for the increase in procedural time. The follow-up and recurrence data are divided temporally between the cohorts, with all the cases after 2018 (except for 7 cases in late 2018) having had sinus mapping completed. Therefore, the follow-up duration was longer for the non–sinus mapping group compared to the sinus mapping group, as demonstrated in the Kaplan–Meier curve **(Figure 3)**.

The challenge of successful AVNRT treatment in the electrophysiology laboratory is identifying the precise site for slow pathway modification to minimize the risk of damage to the AV node. Traditionally, anatomic landmarks and electrophysiologic traits of the slow pathway are used to determine the appropriate ablation site. The subsequent absence of dual AV node physiology after ablation is one marker often used to determine procedural success.^13–15^

The addition of computerized voltage mapping to directly visualize a low-voltage bridge augments electrophysiologists’ ability to precisely target the low-voltage bridge and thus modify the slow pathway while also minimizing risks.^13,14,16,17^ However, not all patients have an identifiable low-voltage bridge due to inadequate voltage point density^14^ or insufficient electrograms within the triangle of Koch.^13^ There is also the potential for multiple low-voltage areas to be identified, making it challenging to isolate the best ablation site.^2^

Even with the high success rate of AVNRT ablation, continued improvement of procedural technique is important. Van Aartsen et al.^2^ established a correlation among sinus propagation wave collision, low-voltage bridge areas, and successful ablation sites, suggesting that SPM improves slow-pathway localization in the pediatric population. Our study demonstrates that the incorporation of SPM can improve the AVNRT ablation technique in the pediatric population by reducing the number of ablations performed until success is achieved. Our median number of ablations needed until success and the total number of ablations for the sinus mapping group are comparable to those in the study by Van Aartsen et al.^2^ There was no difference in acute complications between cohorts, though our study was likely underpowered to uncover this. While the total number of ablations performed between the two groups was similar, the sinus mapping group (n = 20) had more successful slow pathway modifications with the first ablation lesion compared to the non–sinus mapping group. This again shows the improved precision of SPM.

Interestingly, the total number of ablations performed and the total cryoablation time were not statistically different between groups. One possible explanation is that the sinus propagation map appropriately identified the slow pathway, but the ablation catheter engaged only part of the pathway. This would lead to an inadequate initial ablation site for complete slow pathway modification, thus necessitating the need for further ablation lesions and cryoablation time. As mapping technology and techniques continue to improve, the total number of ablations and total cryoablation time needed will likely decrease. This study could also have been underpowered to appreciate a statistically significant difference between the two groups.

The additional procedure duration in the SPM group was statistically significant and is likely secondary to the mapping process. Point-by-point mapping with an ablation catheter likely contributed to the increased duration. The use of multi-electrode catheters for SPM would likely decrease the collection time of the mapping points and therefore reduce the total procedural time. Our center is planning to participate in an upcoming multicenter study comparing the use of multi-electrode catheters for SPM in AVNRT patients. However, this would come at an increased expense due to the cost of multi-electrode catheters. Another possible reason for the increased procedural duration could be related to temporary blunt mechanical trauma of the AV node during the SPM process, thus reducing the likelihood of AVNRT inducibility and prolonging the procedure time. This phenomenon has been experienced at our institution anecdotally. However, as cases of self-resolved blunt AV node trauma are not documented, they could not be accounted for in the data analysis. Also, over time, as sinus mapping technology continues to evolve and the acquisition of mapping points improves, it is likely that total procedural times will shorten. The reduction in the total number of ablation lesions should in theory decrease the procedural times as well. Further studies are being done to evaluate the efficiency of point collection for SPM.

Our study is limited to a single-center, retrospective review. While the survival from recurrence data showed no significant difference between the two groups, this study is likely underpowered to appreciate a significant difference given the low incidence of AVNRT recurrence. Also, the follow-up duration of the SPM group was shorter due to the previously stated temporal association of SPM initiation between the two cohorts. In addition, our study did not evaluate patients with congenital heart disease, those who underwent radiofrequency ablation, or those with other arrhythmia substrates. Future studies would be helpful to evaluate the effectiveness of SPM in these other patient populations.

## Conclusion

In conclusion, SPM is an additional tool that electrophysiologists have at their disposal for mapping the slow pathway for AVNRT. SPM lowered the number of ablation lesions needed in pediatric patients with AVNRT until successful slow pathway modification with cryotherapy. Additional time was needed to collect sufficient data points to create the sinus map.

## Figures and Tables

**Table 1:** Demographics Overall and by Use of Sinus Propagation Mapping

Characteristic	Overall N = 103	No SPM N = 51^a^	SPM N = 52^a^	*P* Value^b^
Age at procedure, years	14.0 (12.0–16.0)	14.0 (12.0–16.0)	15.0 (11.0–16.0)	.9
Sex				.7
Female	62 (60%)	32 (63%)	30 (58%)	
Male	41 (40%)	19 (37%)	22 (42%)	
Height, cm	163 (154, 172)	163 (155, 170)	165 (151, 175)	.3
Weight, kg	55 (46, 65)	55 (49, 60)	57 (44, 69)	.2
BSA, m^2^	1.60 (1.42, 1.77)	1.59 (1.50, 1.67)	1.61 (1.37, 1.83)	.2
Physician				.088
#1	44 (43%)	17 (33%)	27 (52%)	
#2	59 (57%)	34 (67%)	25 (48%)	
System				.2
Carto™	16 (16%)	5 (9.8%)	11 (21%)	
Ensite™	87 (84%)	46 (90%)	41 (79%)	

*Abbreviations:* BSA, body surface area; IQR, interquartile range; SPM, sinus propagation mapping. ^a^Statistics presented: median (IQR) or n (%). ^b^Statistical tests performed: Wilcoxon rank-sum test; chi-squared test.

**Table 2:** Procedural Variables Overall and by Use of Sinus Propagation Mapping

Characteristic	Overall N = 103	No SPM N = 51^a^	SPM N = 52^a^	*P* Value^b^
Inducible AVNRT	102 (99%)	51 (100%)	51 (98%)	>.9
Isoproterenol needed to induce	96 (93%)	45 (88%)	51 (98%)	.060
Typical or atypical AVNRT				.6
Atypical	4 (3.9%)	1 (2.0%)	3 (5.8%)	
Typical	99 (96%)	50 (98%)	49 (94%)	
Total mapping points	N/A	N/A	411 (179–581)	
Ablations until success	3.0 (1.0–8.5)	4.0 (2.0–9.5)	2.0 (1.0–5.2)	**.026**
Time to 1st successful lesion, min	65 (43–87)	52 (40–83)	69 (60–90)	**.012**
Total ablations performed	11 (7–16)	11 (7–15)	11 (8–16)	>.9
Total procedure time, min	142 (115–173)	125 (107–164)	153 (134–179)	**.003**
Total cryoablation time, min	30 (24–44)	28 (24–37)	33 (25–46)	.2
Follow-up, days	401 (56.5–476.5)	446 (388–836)	158 (42–407)	**<.001**
Recurrence	9 (8.7%)	5 (9.8%)	4 (7.7%)	.7
Time to recurrence, days	330 (88–573)	498 (330–688)	108 (76–239)	.3

*Abbreviations:* AVNRT, atrioventricular nodal reentrant tachycardia; IQR, interquartile range; N/A, not applicable; SPM, sinus propagation mapping. ^a^Statistics presented: median (IQR) or n (%). ^b^Statistical tests performed: Wilcoxon rank-sum test and chi-squared test. Bold *P* values are statistically significant (*P* < .05).

## References

[1] Page RL, Joglar JA, Caldwell MA (2016). 2015 ACC/AHA/HRS Guideline for the management of adult patients with supraventricular tachycardia: a report of the American College of Cardiology/American Heart Association Task Force on Clinical Practice Guidelines and the Heart Rhythm Society. Circulation.

[2] Van Aartsen A, Law I, Maldonado J, Von Bergen N (2017). Propagation mapping wave collision correlates to the site of successful ablation during voltage mapping in atrioventricular nodal reentry tachycardia. J Innov Card Rhythm Manag.

[3] Philip Saul J, Kanter RJ, Abrams D (2016). PACES/HRS expert consensus statement on the use of catheter ablation in children and patients with congenital heart disease: developed in partnership with the Pediatric and Congenital Electrophysiology Society (PACES) and the Heart Rhythm Society (HRS) Endorsed by the governing bodies of PACES, HRS, the American Academy of Pediatrics (AAP), the American Heart Association (AHA), and the Association for European Pediatric and Congenital Cardiology (AEPC). Heart Rhythm.

[4] Brugada J, Katritsis DG, Arbelo E (2020). 2019 ESC Guidelines for the management of patients with supraventricular tachycardia. The Task Force for the management of patients with supraventricular tachycardia of the European Society of Cardiology (ESC)Developed in collaboration with the Association for European Paediatric and Congenital Cardiology (AEPC). Eur Heart J.

[5] McCammond AN, Balaji S (2012). Management of tachyarrhythmias in children. Curr Treat Options Cardiovasc Med.

[6] Hollanda Oliveira L, Viana MDS, Luize CM (2022). Underuse of catheter ablation as first-line therapy for supraventricular tachycardia. J Am Heart Assoc.

[7] Drago F, Calvieri C, Russo MS (2021). Low-voltage bridge strategy to guide cryoablation of typical and atypical atrioventricular nodal re-entry tachycardia in children: mid-term outcomes in a large cohort of patients. Europace.

[8] Wells P, Dubuc M, Klein GJ (2018). Intracardiac ablation for atrioventricular nodal reentry tachycardia using a 6 mm distal electrode cryoablation catheter: prospective, multicenter, North American study (ICY-AVNRT STUDY). J Cardiovasc Electrophysiol.

[9] Hanninen M, Yeung-Lai-Wah N, Massel D (2013). Cryoablation versus RF ablation for AVNRT: a meta-analysis and systematic review. J Cardiovasc Electrophysiol.

[10] Lee PC, Tai CT, Lin YJ (2007). Noncontact three-dimensional mapping guides catheter ablation of difficult atrioventricular nodal reentrant tachycardia. Int J Cardiol.

[11] Chang BC, Schuessler RB, Stone CM (1990). Computerized activation sequence mapping of the human atrial septum. Ann Thorac Surg.

[12] Zook N, DeBruler K, Ceresnak S (2022). Identifying an appropriate endpoint for cryoablation in children with atrioventricular nodal reentrant tachycardia: is residual slow pathway conduction associated with recurrence?. Hear Rhythm.

[13] Bailin SJ, Korthas MA, Weers NJ, Hoffman CJ (2011). Direct visualization of the slow pathway using voltage gradient mapping: a novel approach for successful ablation of atrioventricular nodal reentry tachycardia. Europace.

[14] Malloy L, Law IH, Von Bergen NH (2014). Voltage mapping for slow-pathway visualization and ablation of atrioventricular nodal reentry tachycardia in pediatric and young adult patients. Pediatr Cardiol.

[15] Jackman WM, Beckman KJ, McClelland JH (1992). Treatment of supraventricular tachycardia due to atrioventricular nodal reentry by radiofrequency catheter ablation of slow-pathway conduction. N Engl J Med.

[16] Drago F, Battipaglia I, Russo MS (2018). Voltage gradient mapping and electrophysiologically guided cryoablation in children with AVNRT. Europace.

[17] Bearl DW, Mill L, Kugler JD, Prusmack JL, Erickson CC (2015). Visualization of atrioventricular nodal reentry tachycardia slow pathways using voltage mapping for pediatric catheter ablation. Congenit Heart Dis.

